# Poorly controlled cholesterol is associated with cognitive impairment in T2DM: a resting-state fMRI study

**DOI:** 10.1186/s12944-015-0046-x

**Published:** 2015-05-21

**Authors:** Wenqing Xia, Bin Zhang, Yang Yang, Pin Wang, Yue Yang, Shaohua Wang

**Affiliations:** Department of Endocrinology, ZhongDa Hospital of Southeast University, No.87 Dingjiaqiao Road, Nanjing, 210009 PR China; Medical school of Southeast University, No.87 Dingjiaqiao Road, Nanjing, 210009 PR China; Center for functional Neuroimaging, University of Pennsylvania, 3710 Hamilton Walk, Philadelphia, PA 19104 USA; Outpatient Depart, Panda Group Community Health Service Centre, No.4 Qingxi Road, Nanjing, 210009 PR China

**Keywords:** Cholesterol, Type 2 diabetes mellitus, Cognitive impairment, Resting-state fMRI, Functional connectivity

## Abstract

**Background:**

Debate remains on whether hypercholesterolemia is associated with cognitive impairment. Hence, we investigated whether poorly controlled cholesterol impairs functional connectivity among patients with type 2 diabetes mellitus (T2DM).

**Methods:**

Resting-state functional connectivity infers to an interregional cooperation characterized by synchronous and low-frequency (<0.08 Hz) fluctuations on blood oxygen level–dependent functional magnetic resonance imaging (fMRI). We used resting-state fMRI to investigate the functional connectivity of 25 T2DM patients with poorly controlled cholesterol, 22 patients with target cholesterol and 26 healthy controls. Further correlation analysis was conducted between the functional connectivity and clinical data as well as neuropsychological tests.

**Results:**

The three groups did not statistically differ in age, sex, education level, body mass index, blood pressure, fasting C-peptides, and triglyceride. Compared with target cholesterol patients, patients with poorly controlled cholesterol showed significantly increased levels of serum cholesterol, low-density lipoprotein (LDL), and LDL/high-density lipoproteins (HDL) ratio, as well as poor performance in Trail Making Test B (TMT-B) (*p* < 0.05). Disordered functional connectivity of bilateral hippocampus-middle frontal gyrus (MFG) in the poorly controlled group consistently existed when compared with the two other groups. Moreover, the aberrant functional connectivity was associated with the TMT-B scores and the LDL/HDL index in T2DM patients with poorly controlled cholesterol.

**Conclusions:**

T2DM patients with poorly controlled cholesterol showed impaired attention and executive function. The resting-state connectivity disturbance of the hippocampus-MFG may be involved in this process. Decreasing the LDL/HDL ratio can be taken as precaution against cognitive decrements.

## Background

Numerous publications support that type 2 diabetes mellitus (T2DM) patients have an increased risk of cognitive impairment, especially learning and memory deficits [1, 2]. Diabetes mellitus (DM) is associated with a 1.5-fold to 2.0-fold increased risk of Alzheimer’s disease (AD), as well as 2.0-fold to 2.5-fold increased risk of vascular dementia [3]. In AD, the hippocampus is one of the primary brain regions suffering damage, with early symptoms including memory loss and disorientation [4, 5]. Furthermore, T2DM patients suffer from specific impairment in the function and structure of the hippocampus [6–8].

Debate remains on whether hypercholesterolemia, which often exists in T2DM patients, is etiologically associated with cognitive impairment or dementia. Although prior researches have failed to confirm that elevated cholesterol brings a risk factor in developing AD [9, 10], most studies found a positive relationship between elevated cholesterol and cognitive impairment [11–13]. Various studies have also focused on the relationship between cognition and high-density lipoprotein (HDL) and low-density lipoprotein (LDL) [14, 15]. To date, few studies on their effects on cognitive impairment in T2DM patients reported conflicting results. For instance, a recent 40-month longitudinal study suggested that LDL level control was ineffective in reducing cognitive decline among persons with poorly controlled T2DM [16]. By contrast, two Asian research groups found significant correlations between cognitive impairment and abnormal levels of HDL and LDL in T2DM patients [17–19]. However, all the above studies evaluated the cognitive state of individuals by performing neuropsychological tests, which can only provide some rough information on relevant domains of cognition rather than specific brain areas. Thus, the relationship between diabetes-related cognitive impairment and abnormal cholesterol remains to be elucidated.

Traditional epidemiological studies have provided abundant evidence regarding cognitive decline in T2DM patients. As a complement to them, the resting-state functional magnetic resonance imaging (rs-fMRI) method is typically introduced to investigate changes in the brain of T2DM patients. Our previous work confirmed that T2DM patients show altered amplitude of low-frequency fluctuations in many brain regions, indicating that fMRI detects altered baseline brain activity in patients with T2DM. Our work also concluded that the abnormal alterations in the middle temporal gyrus may play a central role in T2DM-related cognitive decline [20]. Another fMRI study also observed that the hippocampus displayed bilaterally decreased functional connectivity to widespread regions in T2DM patients [21].

Resting-state functional connectivity infers to an interregional cooperation, which can be characterized by synchronous and low-frequency (<0.08 Hz) fluctuations on blood oxygen level dependent fMRI [22]. To our knowledge, no research has focused on the influence of poor cholesterol control on the cognitive function of diabetes patients by using fMRI. Hence, the objective of this study is to investigate whether increased cholesterol is related to the decline in functional connectivity between the bilateral hippocampus and other brain regions. And, if so, we further examine whether this altered functional connectivity is correlated with clinical information and neurocognitive performance in T2DM patients.

## Results and discussion

### Clinical and neuropsychological data

Participant characteristics, clinical ratings, and corrected neuropsychological scores are listed in Table 1. The three groups did not statistically differ in age, sex, education level, body mass index (BMI), blood pressure, fasting C-peptides, triglyceride, intima-media thickness, presence of WMH and lacunar infarcts. In terms of neuropsychological tests, only Trail Making Test B (TMT-B) (*p* < 0.05) showed significant differences in the three groups. Overall, the poorly controlled cholesterol group performed the worst. Compared with target cholesterol patients, patients with poorly controlled cholesterol showed significantly increased levels of serum cholesterol, LDL, and LDL/HDL index, as well as poor performance in TMT-B (*p* < 0.05).

**Table 1:** Demographic, clinical, and cognitive characteristics

	T2DM patients with higher cholesterol (n = 25)	T2DM patients with lower cholesterol (n = 22)	Healthy controls (n = 26)	*p*-value (ANOVA)
Age (year)	58.1 ± 8.1	57.7 ± 7.6	55.1 ± 6.5	0.293
Gender (male:female)	13:12	10:12	13:13	0.835
Education levels (years)	9.6 ± 3.8	10.8 ± 3.2	10.5 ± 2.6	0.443
Diabetes duration (years)	10.4 ± 5.8	9.4 ± 6.3	--	0.565
BMI (kg/m^2^)	24.6 ± 2.9	24.1 ± 2.6	23.8 ± 2.8	0.605
Waist-hip ratio	0.9 ± 0.1	0.9 ± 0.1	0.8 ± 0.1	0.4640.810
Systolic BP (mmHg)	133.6 ± 13.5	130.1 ± 12.5	127.5 ± 13.9	0.357
Diastolic BP (mmHg)	81.1 ± 8.8	80.7 ± 6.8	83.1 ± 8.4	0.627
Hb_A1c_ (%)	8.3 ± 1.7	7.2 ± 0.7	5.3 ± 0.3	<0.001*
Fasting glucose (mmol/L)	8.3 ± 2.2	7.4 ± 1.1	5.4 ± 0.3	<0.001*
Glucose after 2-h oral glucose tolerance test (mmol/l)	15.5 ± 5.4	14.0 ± 3.1	5.8 ± 1.0	<0.001*
Fasting C-peptide(ng/ml)	2.0 ± 1.0	1.5 ± 0.5	2.0 ± 0.9	0.135
Triglyceride (mg/dl)	159.4 ± 141.7	115.1 ± 106.3	124.0 ± 70.9	0.339
Total cholesterol (mg/dl)	224.3 ± 42.5^a^	166.3 ± 19.3	193.3 ± 19.3	<0.001*
LDL (mg/dl)	139.2 ± 23.2^a^	92.8 ± 11.6	112.1 ± 19.3	<0.001*
HDL (mg/dl)	54.1 ± 11.6	50.3 ± 11.6	54.1 ± 7.7	0.871
LDL/HDL index	2.8 ± 0.6	1.9 ± 0.5	2.1 ± 0.5	<0.001*
White matter hyperintensity^b^	0(0-5)	0(0-6)	0(0-4)	0.498
Lacunar infarcts (yes: no)^c^	5:20	4:18	4:22	0.913
Intima-mediathickness, mm	0.9 ± 0.2	1.0 ± 0.2	0.9 ± 0.2	0.359
MMSE	28.0 ± 0.3	28.7 ± 0.5	28.9 ± 0.6	0.350
MoCA	22.4 ± 0.8	23.1 ± 1.1	24.7 ± 1.3	0.421
AVLT	31.3 ± 2.1	27.5 ± 3.0	36.8 ± 3.6	0.092
CFT	34.7 ± 0.4	35.3 ± 0.6	34.4 ± 0.7	0.581
CFT- delayed recall	15.5 ± 1.4	14.6 ± 2.0	16.1 ± 2.4	0.873
TMT-A	71.2 ± 5.7	66.0 ± 8.0	64.2 ± 9.7	0.821
TMT-B	241.2 ± 17.2^a^	164.0 ± 24.2	131.9 ± 29.0	0.011*
CDT	3.2 ± 0.2	3.2 ± 0.2	3.3 ± 0.3	0.929
DST	10.6 ± 0.5	12.0 ± 0.8	13.0 ± 0.9	0.176
VFT	18.0 ± 1.1	19.3 ± 1.6	18.4 ± 1.9	0.810

* Indicated significant differences among the three groups (*p* <0.05)

^a^ Indicated significant differences in clinical data and cognitive performances between T2DM patients with target cholesterol level and T2DM patients with poorly controlled cholesterol (*p* <0.05)

^b^ White matter hyperintensity were defined as ill-defined hyperintensities ≥ 5 mm on both T2 and FLAIR images

^c^ Lacunar infarcts were defined as well-defined areas of > 2 mm with signal characteristics on MRI the same as cerebrospinal fluid

For TMT-A and B, a higher score corresponds to a poorer performance, whereas for the rest of the cognitive tests, a higher score corresponds to a better performance

Abbreviations: LDL-C, low-density lipoprotein cholesterol; HDL-C, high-density lipoprotein cholesterol; MMSE, Mini Mental State Exam; MoCA, Montreal Cognitive Assessment; AVLT, Auditory Verbal Learning test; CFT, Rey-Osterreith Complex Figure Test; TMT, Trail Making Test; CDT, Clock Drawing Test; DST, Digit Span Test; VFT, Verbal fluency test

### Functional connectivity data

**Fig. 1:**
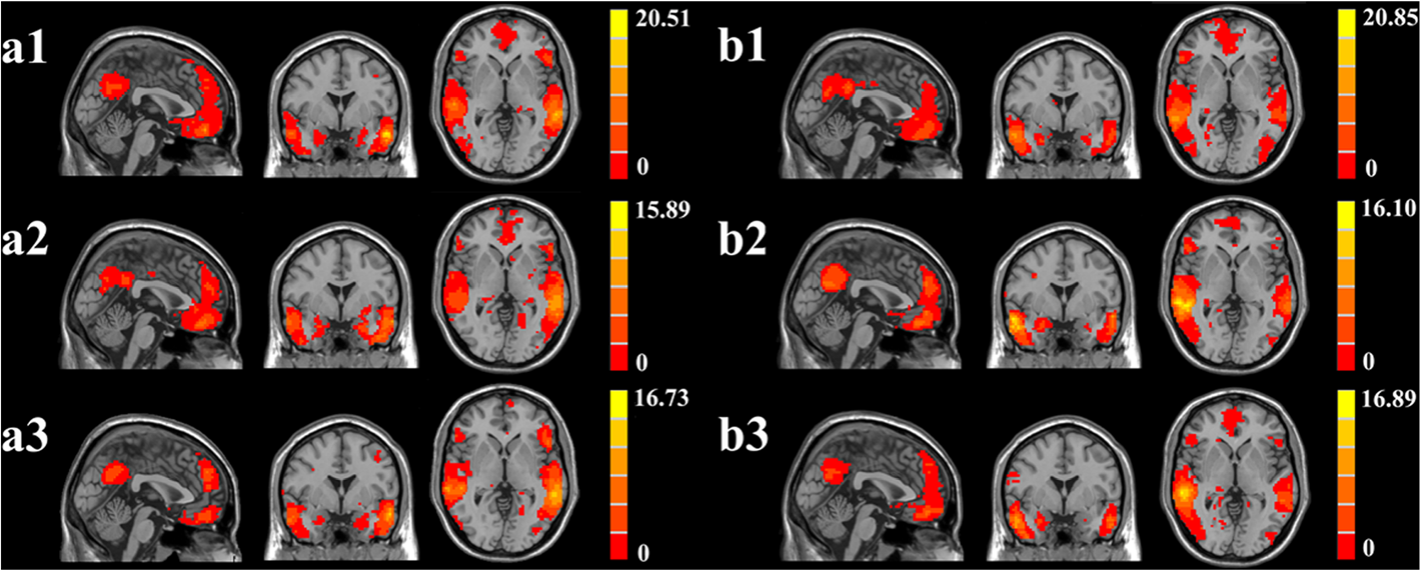
Significant brain functional connectivity to the bilateral hippocampus using one-sample *t*-test: (a1) left hippocampal functional connectivity for healthy controls; (a2) left hippocampal functional connectivity for T2DM patients with target cholesterol; (a3) left hippocampal functional connectivity for T2DM patients with poorly controlled cholesterol; (b1) right hippocampal functional connectivity for healthy controls; (b2) right hippocampal functional connectivity for T2DM patients with target cholesterol; and (b3) right hippocampal functional connectivity for T2DM patients with poorly controlled cholesterol. Thresholds were set at a corrected *p* < 0.05, determined by Monte Carlo simulation. The left side corresponds to the right hemisphere of the brain

A one-sample *t*-test revealed that the bilateral hippocampus in all the groups demonstrated strong connectivity to other brain regions, such as the anterior cingulate cortex, posterior cingulate cortex, inferior parietal lobule (IPL), middle frontal gyrus (MFG), middle temporal gyrus, and precuneus. The results were consistent with the default mode network (DMN) proposed by Raichle *et al.* [23]. Similar changes were also observed in several other regions, such as the cerebellum posterior lobe, parahippocampal gyrus, and occipital middle lobe (Fig. 1).

**Fig. 2:**
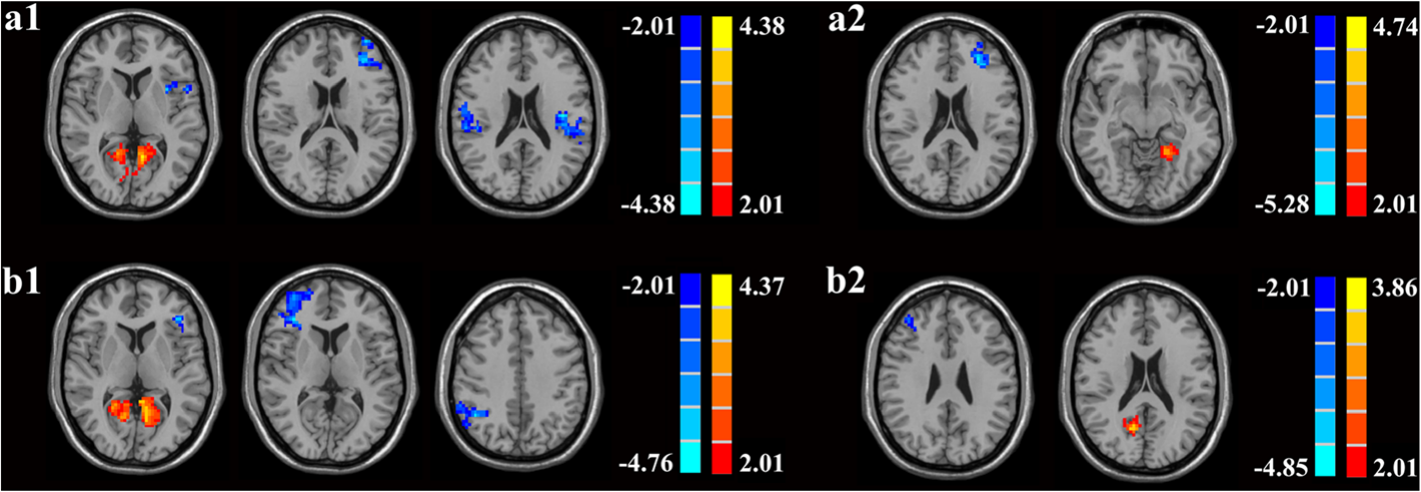
Results of post-hoc analysis. (a1) For the left hippocampus, T2DM patients with poorly controlled cholesterol had decreased functional connectivity in the left insula, left MFG, and bilateral IPL and increased connectivity was seen in the bilateral calcarine and left lingual gyrus compared with healthy controls; (a2) For the left hippocampus, T2DM patients with poorly controlled cholesterol had decreased connectivity in the left MFG and increased connectivity in the left lingual gyrus when compared with T2DM patients with target cholesterol; (b1) For the right hippocampus, T2DM patients with poorly controlled cholesterol had decreased connectivity in the left IFG, right MFG, and right IPL and their bilateral calcarine and right lingual had increased functional connectivity compared with the healthy controls; (b2) For the right hippocampus, the poorly controlled cholesterol group showed decreased functional connectivity in the right MFG and increased functional connectivity in the right precuneus compared with the target cholesterol group. Thresholds were set at a corrected *p* < 0.05, determined by Monte Carlo simulation. The left side corresponds to the right hemisphere of the brain

One-way ANOVA showed significant differences in the functional connectivity of the left hippocampus among the three groups; the regions included the left insula, left MFG, bilateral IPL, bilateral calcarine, and left lingual gyrus. Significant differences also exised in the right hippocampus among the three groups; the regions included the left IFG, right MFG, right IPL, bilateral calcarine, right lingual, and right precuneus. Compared with healthy controls, patients with poorly controlled cholesterol showed decreased functional connectivity in the following brain regions: left insula, left MFG and bilateral IPL. An increased connectivity was observed in the bilateral calcarine and left lingual gyrus (Fig.2a1 and Table 2). Furthermore, patients with poorly controlled cholesterol showed decreased connectivity in the left MFG and increased connectivity in the left lingual gyrus compared with target cholesterol patients (Fig. 2a2 and Table 2). Fig. 2b1 and Table 2 show the different connectivities in the right hippocampus. Patients with poorly controlled cholesterol exhibited decreased connectivity in the left IFG, right MFG, and right IPL. Patients with poorly controlled cholesterol also demonstrated increased functional connectivity in the bilateral calcarine and right lingual compared with the healthy controls. Compared with the target cholesterol group, the poorly controlled cholesterol group showed decreased functional connectivity in the right MFG and increased functional connectivity in the right precuneus (Fig. 2b2 and Table 2).

**Table 2:** Regions showing significant functional connectivity differences on hippocampus of T2DM patients group compared with healthy controls

Brain regions	BA	MNI Coordinates x, y, z (mm)	Peak t score	Voxels
**A.** Differences on left hippocampus				
(I) T2DM patients with poorly controlled cholesterol versus with healthy controls				
Decreased in T2DM patients with poorly controlled cholesterol				
L insula	13	−33,9,6	−3.5398	131
L middle frontal gyrus	10	−36,57,18	−3.6381	291
L inferior parietal lobule	39/40	−39,-15,21	−3.9687	220
R inferior parietal lobule	39/40	45,-33,27	−4.0773	130
Increased in T2DM patients with poorly controlled cholesterol				
L calcarine/ L lingual gyrus	17	−9,-57,9	4.3789	552
R calcarine	17	9,-96,-3	3.4545	124
(II) T2DM patients with poorly controlled cholesterol versus with T2DM patients with target cholesterol levels				
Decreased in T2DM patients with poorly controlled cholesterol				
L middle frontal gyrus	46	−27,42,21	−5.2780	195
Increased in T2DM patients with poorly controlled cholesterol				
L lingual gyrus	17	−21,-51,-9	3.6838	85
**B.** Differences on right hippocampus				
(I) T2DM patients with poorly controlled cholesterol versus with healthy controls				
Decreased in T2DM patients with poorly controlled cholesterol				
L inferior frontal gyrus	44/45/46/47	−39,33,6	−4.2669	198
R middle frontal gyrus	46	36,33,9	−4.7610	1281
R inferior parietal lobule	40	45,-54,39	−4.5285	259
Increased in T2DM patients with poorly controlled cholesterol				
R calcarine/ R lingual	17	9,-60,0	4.3660	256
L calcarine	17	−9,-57,6	3.9207	289
(II) T2DM patients with poorly controlled cholesterol versus with patients with target cholesterol levels				
Decreased in T2DM patients with poorly controlled cholesterol				
R middle frontal gyrus	46	36,39,27	−3.9874	267
Increased in T2DM patients with poorly controlled cholesterol				
R precuneus	7	12,-63,21	3.4747	145

A corrected threshold of *p* < 0.05 determined by Monte Carlo simulation was taken as meaning that there was a significant difference between groups. BA, Brodmann’s area; MNI: Montreal Neurological Institute; L, left; R, right; B = bilateral; cluster size is in mm^3^

### Correlation analysis results

**Fig. 3:**
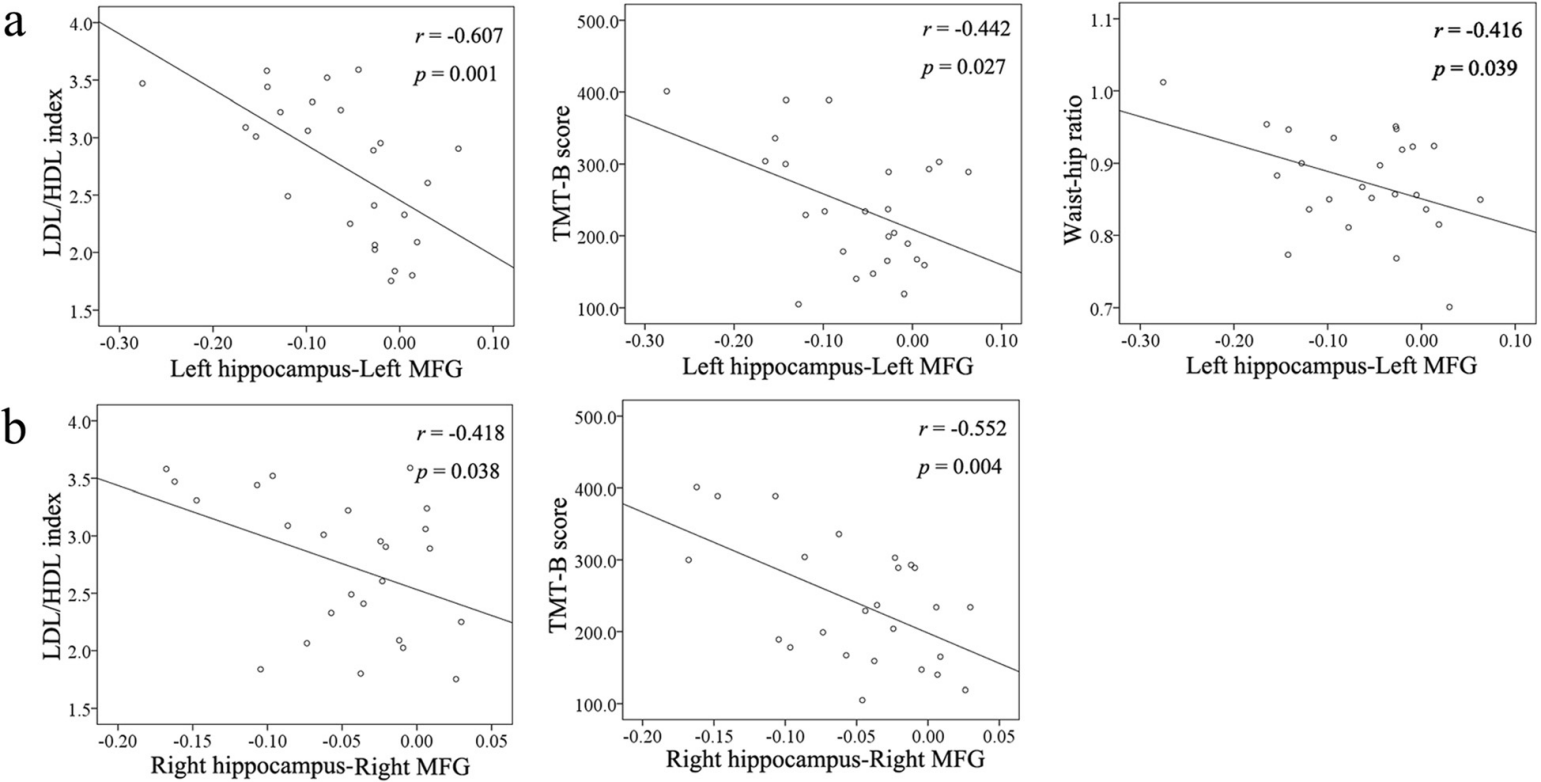
**a** Correlation between clinical data (low-density lipoproteins (LDL)/high-density lipoproteins (HDL) ratio, TMT-B scores, and waist-hip ratio (WHR)) and the functional connectivity of the left hippocampus-left MFG. **b** Correlation between clinical data (LDL/HDL index, TMT-B scores, and WHR) and the functional connectivity of the right hippocampus-right MFG

Patients with poorly controlled cholesterol showed significant correlations between the clinical data (LDL/HDL ratio, TMT-B scores, and waist-hip ratio (WHR)) and the functional connectivity of the left hippocampus-left MFG (*r* = −0.607, *p* = 0.001; *r* = −0.442, *p* = 0.027; *r* = −0.416, *p* = 0.039, respectively) (Fig. 3a), as well as significant correlations between the clinical data (LDL/HDL ratio and TMT-B scores) and the functional connectivity of the right hippocampus-right MFG (*r* = −0.418, *p* = 0.038; *r* = −0.552, *p* = 0.004, respectively) (Fig. 3b). By contrast, no significant correlations existed between the WHR and the functional connectivity between right hippocampus and the right MFG.

## Discussion

Implications of previous studies focusing on the relationship between dyslipidemia and cognitive impairment remain controversial. In the current study, fMRI methods were used to provide initial support for the deleterious effect of elevated cholesterol on attention and executive function of T2DM patients. Correlation between the rs-fMRI information and clinical data also suggested that functional connectivity disturbance of the hippocampus-MFG may occupy an important status in the cognitive dysfunction of T2DM patients who failed to meet the cholesterol targets.

In this study, multidimensional neuropsychological tests were conducted to evaluate individual cognitive function. Global cognitive function was evaluated using Mini Mental State Exam (MMSE) and Montreal Cognitive Assessment (MoCA). MMSE is the most widely used test to assess dementia, which focuses on five different cognitive domains, namely, orientation, immediate memory, delayed memory, attention/calculation, and linguistic capacity. MoCA is developed as a brief screening instrument for MCI and is more sensitive than MMSE. Memory decline was revealed by Digit Span Test (DST), Auditory Verbal Learning Test (AVLT), Verbal Fluency Test (VFT), and Rey-Osterreith Complex Figure Test (CFT) -delay. CFT-copy and Clock Drawing Test (CDT) assessed the domain of visuoconstruction. Trail Making Test-A (TMT-A) reflected the information-processing speed. However, all the above tests showed insignificant differences among the three groups. These results may partially be attributed to the relatively younger ages and shorter disease durations. In addition, most neuropsychological tests cannot detect subtle cognitive changes in such limited population. Nevertheless, the diabetic patients, particularly the poorly controlled group, showed slight decline of scores in most of the tests. These changes revealed that patients with increased cholesterol suffered cognitive decline in certain aspects. Instructively, the TMT-B score in the poorly controlled group showed significantly decreasing trends compared with target cholesterol patients. In this study, the deficits of attention and executive function defined by TMT-B were the most prominent characteristics of the T2DM patients with poorly controlled cholesterol. This result specifically indicated that uncontrollable cholesterol contributed overt damage to the attention and executive function of T2DM patients.

One-sample *t*-tests showed an increased functional connectivity within the DMN in each group, which corresponded well with the consensus that the DMN is involved in maintaining baseline brain activities [23]. Similarly, a decline in functional connectivity was observed in the DMN of patients with poorly controlled cholesterol compared with the control subjects. To some extent, the results were in agreement with previous research [24]. The primary finding of the present study indicated that the poorly controlled group exhibited disrupted functional connectivity in the MFG compared with target cholesterol patients. By contrast, several distributed brain regions, including the calcarine, lingual gyrus, and precuneus, showed enhanced functional connectivity, indicating that some compensatory mechanisms may overcome the breakdown of communication in other brain areas. Among these selected brain regions, the MFG has elicited considerable interest, for its disordered functional connectivity in the poor controlled group had been consistently shown when compared with the two other groups. Coincidentally, as a commonly used neuropsychological test that reflects frontal functioning, TMT-B scores were paralleled with functional connectivity measures in the MFG. These scores suggested that the depressed functional connectivity, which implies the impaired MFG function, may be vital in identifying the existence of cholesterol-related cognitive dysfunction in T2DM patients.

Interestingly, the present study also found negative correlations between the functional connectivity measures in the MFG of patients with poorly controlled cholesterol and their clinical indices, including LDL/HDL ratio and WHR. Abnormal lipid levels showed undoubtedly significant contribution to the risk of coronary heart disease. Theoretically, dyslipidemia increases the incidence rate of cerebrovascular disease, which is also related to the risk of dementia [25]. High cholesterol also plays a predominant role in the development of atherosclerosis and coronary heart disease [26]. Thus, high cholesterol level may be a modifiable risk factor for future cognitive decline and dementia. In older adults, atherosclerosis coincides with poorer attention and executive function [27], a finding partially in line with our results. The present study suggested that the LDL/HDL ratio, as a strong predictor of cardiac events, may be a predictive factor of cholesterol-related cognitive decline in T2DM to a certain extent. Obesity, particularly mid-life obesity, has been determined to be associated with increased risk of cognitive impairment and dementia [28]. Central adiposity estimated by higher WHRs is associated with decreased cognitive function scores [29]. Previous studies have demonstrated that higher WHR, which indicates critical resources for brain development, are associated with poorer cognitive performance and detrimental changes in the brain [30–33]. Similar to LDL/HDL ratio, WHR is also a cardiovascular risk factor in the elderly. However, this study failed to detect the correlation between functional connectivity of the right hippocampus-MFG and WHR. We infer that computed tomography and MRI scan should be further used to assess visceral fat deposition, which significantly influences on the WHR.

Certain limitations should be noted in the current study. First, as a preliminary study, the cross-section study showed a relatively small patient cohort. The number of participants in this study is based on the sample size in previous literature. So far, most previous fMRI studies on T2DM had small sample size, ranging from 10 to 30 subjects in each group [34–36]. Although based on relatively small sample size, not only these previous rs-fMRI studies but our study was able to obtained significant findings. As fMRI results were obtained after strict multiple comparison correction, which guaranteed the credibility of these significant results, it implied that the rs-fMRI is a sensitive tool for detecting the subtle changes in brain activity and neural network. Nevertheless, the small sample size is still a major limitation, leading to aberrant findings such as the low LDL level this limited sample size may lead to low levels of LDL in patients with well-regulated cholesterol and relatively lower triglyceride level in all patients. In addition, although we expected to focus on LDL, avoiding the potential effects of other kinds of cholesterol (*e.g.*, triglyceride and HDL), was difficult because we cannot perform further stratification based on the current population. Thus, enlarging the sample size and following up on these patients are necessary to examine whether the disturbed functional connectivity in the hippocampus links to the abnormal cholesterol level. Second, the average cholesterol of the healthy controls was slightly higher than that of the T2DM patients. This average can be ascribed to the differences in the grouping criteria for cholesterol: ADA for patients and normal range in our hospital for healthy controls. Finally, multiple metabolic factors, such as glucose and C-peptide, are involved in the development of cognitive decline in T2DM. Although the two diabetic groups showed similar clinical characteristics at baseline because of the strict inclusion criteria, we added these possible confounders as covariates to eliminate the interference to some extent, and more in-depth studies are required in this field to confirm the conclusions.

## Conclusions

Despite of the limitations, our findings provide clinical implications. Functional connectivity was used in the current investigation to prove the effects of poorly controlled cholesterol on the attention and executive function of T2DM patients. The results also proved that the resting-state connectivity disturbance of the hippocampus-MFG may play a significant role in the cognitive dysfunction of T2DM with poorly controlled cholesterol levels. The cross-sectional findings also provide information on the role of decreasing LDL/HDL ratio to ameliorate the cognitive decrements. Thus, the present findings offer some instructive information regarding the intervention of cognitive decrements in T2DM patients to some extent. Nevertheless, more comprehensive research, especially longitudinal studies, should be performed to confirm these implications.

## Methods

### Subjects

A total of 80 right-handed subjects including 52 T2DM patients (aged between 45 and 70 years) and 28 healthy controls were recruited *via* normal community health screening and from a hospital from September 2012 to September 2013. T2DM diagnosis was based on the World Health Organization (1999) criteria [37]. In accordance with the lipid control goal of the American Diabetes Association (ADA) [38], patients with LDL-C > 2.6 mmol/l or HDL-C < 1.0 mmol/l for men and < 1.3 mmol/l for women were assigned to the poorly controlled cholesterol group (n = 27). The rest was classified into the target cholesterol group (n = 25). No patients had consumed any cholesterol-lowering drugs, owing to treatment cost and other reasons. Healthy controls were recruited from the community during the same period the study was conducted.

To eliminate the potential factor that may affect the results of this study, we excluded those patients with very high glucose levels (fasting glucose > 10 mmol/l, postprandial glucose > 20 mmol/l, HbA1c > 10 %). Participants were excluded from the study if they declared a history of known stroke, alcoholism, head injury, Parkinson’s disease, epilepsy, major depression, other acute neurological or psychiatric illnesses, major medical illnesses (*e.g.*, cancer, anemia, thyroid dysfunction, severe heart diseases and damaged liver or kidney function), and severe visual or hearing loss.

The protocol and informed consent documents were approved by the Research Ethics Committee of the Affiliated Zhongda Hospital of Southeast University. All participants provided written informed consent prior to each assessment.

### Clinical data and neuropsychological test information

The study followed a cross-sectional design. Demographic characteristics were obtained. Blood samples were collected twice by venipuncture at 8 A.M. after an overnight fast and 10 A.M. after drinking a 75 g glucose solution to measure the levels of fasting blood glucose, fasting serum C-peptide, HbA1c, triglyceride, total cholesterol, LDL-C, HDL-C, and glucose after 2-h oral glucose tolerance test.

A battery of neuropsychological tests was administrated to all the subjects to evaluate individual neuropsychological status [21, 39]. MMSE was used to assess possible dementia. MoCA was used to evaluate general cognitive status. AVLT and CFT were used to reveal episodic memory. DST, TMT-A and TMT-B were used to assess attention and psychomotor speed. CDT was used to reflect visuospatial function, and VFT was used to examine semantic memory. For TMT-A and TMT-B, a higher score corresponds to a poorer performance, whereas for the rest of the tests, a higher score corresponds to a better performance. A neuropsychiatry specialist facilitated the process by using a single-blind method. None of the participants displayed audiovisual or motor coordination impairment that can affect the neuropsychological tests.

### Data acquisition

Subjects were scanned using a 3.0 T MRI scanner (Siemens MAGENETOM Trio) with a birdcage head coil. Subjects were laid supine with their head fixed by foam pads and a belt to minimize head motion. Earplugs were given to the subjects to reduce scanner noise. The subjects were instructed to lie quietly with their eyes closed but not to fall asleep, not to think of anything in particular, and to avoid head motion during fMRI. All participants underwent the scan at daytime with proper compliance.

Functional images were collected axially by using an echo-planar imaging (EPI) sequence as follows: repetition time (TR) = 2000 ms, echo time (TE) = 25 ms, slices = 36, thickness = 4 mm, gap = 0 mm, field of view (FOV) = 240 × 240 mm^2^, acquisition matrix = 64 × 64, and flip angle (FA) = 90°. High-resolution 3D T1-weighted axial images covering the entire brain were acquired using the following parameters: TR = 1900 ms, TE = 2.48 ms, slices = 176, thickness = 1 mm, gap = 0 mm, FA = 90°, acquisition matrix = 256 × 256, FOV = 250 × 250 mm^2^. The entire process lasted for 12 min and 24 s.

On the basis of the age-related white matter changes scale [40], the assessment of white matter hyperintensity (WMH) and lacunar infarcts were quantitatively evaluated on the fluid-attenuated inversion recovery images by experienced radiologists. A single-blind method was also used. Participants with a rating score above one were excluded.

### Image preprocessing

Analyses were conducted with Data Processing Assistant for rs-fMRI (DPARSF) programs [41], which are based on statistical parametric mapping (SPM8, http://www.fil.ion.ucl.ac.uk/spm) and rs-fMRI data analysis toolkits (REST, http://www.restfmri.net).

As described in our previous work [20], the first ten volumes were discarded, considering the factors that signal equilibrium of the initial MR signals and the adaptation of the subjects to the circumstances. Then the remaining 230 consecutive volumes were used for data analysis. Subsequently, we performed the following in order: slice-timing adjustment, realignment for head-motion correction, spatial normalization to the Montreal Neurological Institute (MNI) template (resampling voxel size = 3 × 3 × 3 mm^3^), smoothing with an isotropic Gaussian kernel (FWHM = 4 mm), detrend and filtering (0.01–0.08 Hz).

We excluded the following from the study: one healthy control with poor quality of image and seven participants (two patients with higher cholesterol, three patients with lower cholesterol, and two healthy controls) with head movement exceeding 2.0 mm of maximum translation in any of the x, y, and z directions or 2.0° of the maximum rotation about the three axes. We further analyzed 25 patients with higher cholesterol, 22 patients with lower cholesterol, and 26 healthy controls.

### Functional connectivity

Functional connectivity analyses were performed using REST software. The seed region of interests (ROIs) of the bilateral hippocampus were generated using the WFU_PickAtlas Tool v.2.4 software (http://www.ansir.wfubmc.edu). For each subject, the mean time series for the ROIs was calculated as the reference time course. Then, temporal correlation analysis was performed between the mean signal changes of ROI and time series of the remaining brain areas. Finally, a Fisher’s r-to-z transform was applied to improve normality of the correlation coefficients [42]. Six head motion parameters, the mean time series of global white matter, and cerebrospinal fluid signals were introduced as covariates to remove random effects in the results.

### Statistical analysis

#### Demographic and clinical characteristics analysis

Differences in demographic information and clinic measures among groups were analyzed using ANOVA first among the three groups followed by a post hoc test (*t*-test for means and χ^*2*^-test for proportions) between T2DM patients with target cholesterol and T2DM patients with uncontrolled cholesterol. A general linear model was used to test for differences in neuropsychological test scores to control HbA1c and fasting glucose levels to avoid a bias effect of these variables. Thresholds were set at a corrected *p* < 0.05.

#### Functional connectivity analysis

One-sample *t*-test was performed on the individual functional connectivity maps in a voxel-wise manner. The test was used to determine the patterns of functional connectivity of regions with significant and positive connectivity to the specific seed in each group. One-way analysis of variance (ANOVA) was then performed to determine the functional connectivity differences among the three groups. To explore the between-group differences in functional connectivity, post hoc analysis was further conducted by one-way ANOVA, with age, sex, education, head motion, fasting C-peptide, BMI, triglyceride, HbA1c, fasting and postprandial glucose levels, intima-mediathickness, and presence of WMH and lacunar infarcts importing as nuisance covariates to control for the influences of these factors on the results. Thresholds were all set at a corrected *p* < 0.05, with multiple comparison correction using AlphaSim program (http://afni.nimh.nih.gov/pub/dist/doc/manual/AlphaSim.pdf) and determined by Monte Carlo simulation. The parameters were single voxel *p* value = 0.05, a minimum cluster size of 85 mm^3^, and FWHM = 4 mm within a gray matter mask corresponding to Automated Analytical Labeling AAL atlas [43].

#### Correlation analysis

The mean z-values were extracted to investigate the association between functional connectivity of hippocampus-MFG and clinical data and neurocognitive performances of T2DM patients with higher cholesterol. Then, the Pearson’s correlation coefficients between the clinical data, results of each neuropsychiatric test and the individual z-values in patients with higher cholesterol group were analyzed by SPSS software (version 17.0). A value of *p* < 0.05 was considered statistically significant. The correlations were also corrected for age, sex and other possible confounders mentioned before.
